# Functional Mobility Assessment in People with Multiple Sclerosis

**DOI:** 10.3390/neurolint17050063

**Published:** 2025-04-23

**Authors:** Ivana Sretenović, Srećko Potić, Goran Nedović, Gordana Odović, Ljiljana Šimpraga

**Affiliations:** 1 Faculty of Special Education and Rehabilitation, University of Belgrade,11000 Belgrade, Serbia; 2 High Medical College of Professional Studies “Milutin Milanković”, 11000 Belgrade, Serbia; 3 Academy of Applied Studies Belgrade, The College of Health Sciences, 11000 Belgrade, Serbia

**Keywords:** balance, walking, multiple sclerosis, patient-reported outcomes

## Abstract

Background/Objectives: Functional mobility includes gait and balance. People with multiple sclerosis often experience gait impairment and difficulties with walking, as well as an increased risk of falling. The aim of the research was to assess functional mobility and to examine the relationship between gait and balance in people with multiple sclerosis, as well as the impact of falls on these two variables. Methods: The study sample consisted of 92 people with multiple sclerosis, with an average age of 45.10 (SD = 9.57) years, and both sexes (82.6% were female). The Activities-specific Balance Confidence Scale was used to assess an individual’s confidence in maintaining balance throughout daily activities, and the 12-item Multiple Sclerosis Walking Scale was employed to evaluate the impact of multiple sclerosis on walking ability. Descriptive statistics, measures of central tendency, Pearson’s correlations, and partial correlations were applied to the data. Results: The results indicated moderate gait impairment and a high level of function in people with multiple sclerosis. There was a correlation between confidence in maintaining balance and walking ability. Conclusions: The results of this study can be used to develop appropriate treatments and support programs for individuals with multiple sclerosis.

## 1. Introduction

Multiple sclerosis (MS) is an inflammatory and neurodegenerative disease of the central nervous system [1], classified among chronic and incurable neurological diseases, with approximately 11.972 people diagnosed in the Republic of Serbia in 2024 [2] and an estimated 2.9 million people with multiple sclerosis (pwMS) worldwide [3]. MS is more common in women than in men, with reported female-to-male ratios of 3:1 [4] and 4:1 [5]. The disease occurs between the ages of 20 and 40 years [4].

Despite the fact that the pathogenesis of MS remains uncertain, motor, sensory, visual, and autonomic systems are affected [6]. Damage to the brain and spinal cord leads to a wide range of symptoms that impact the motor, cognitive, social, and emotional functioning of these individuals [7].

Research suggests that men and women experience different MS symptoms. Women with MS often report more fatigue, pain, and psychiatric symptoms (like depression and anxiety) compared to men [8]. These factors can severely impact the quality of life and contribute to disability, even if the physical disability from MS appears less severe. Men with MS may experience more motor-related impairments, including greater muscle weakness, gait disturbances, and coordination problems [9]. These symptoms often manifest earlier in the disease course and tend to progress more severely over time [10]. MS symptoms vary considerably among individuals. However, motor impairments are among the most prevalent manifestations, with the majority of pwMS experiencing gait disturbances and walking difficulties as the disease progresses [11]. Gait and walking are essential components of functional mobility, making their assessment a crucial aspect of patient monitoring in clinical practice and research contexts. Changes in functional mobility are among the most common and disabling consequences of MS [12]. Between 70 and 75% of pwMS have been reported to have gait problems [13]. Gait problems are observed mildly in individuals with low disability levels (Expanded Disability Status Scale-EDSS: 0–1.5) among those in the early stages of the disease [11]. However, walking capacity tends to deteriorate with time [14]. Impairment of mobility due to the progression of MS has a significant impact on functional independence and quality of life of people with MS [15]. Additionally, deficits in mobility, balance, and coordination often contribute to physical disabilities that can progressively worsen in individuals with MS.

In addition to problems with gait, movement, and balance, more than 63% of pwMS report experiencing at least one fall within a two- to six-month period [15,16]. Impaired gait, balance, and mobility increase the risk of falls in pwMS [17,18,19], with the majority of falls occurring during transfers, ambulation, standing, navigating stairs or curbs, and exercising or engaging in physical activity [20].

The most commonly self-reported causes of falls include tripping or slipping, exhaustion, inattention, haste, failure to use assistive devices, dizziness, carrying objects, and visual impairment [20].

A study on patients with relapsing-remitting MS found that 77% experienced balance difficulties, which reduced mobility and increased the risk of falling. These problems negatively impact patients′ daily activities and emotional well-being [21].

Therefore, the aim of the research was to assess functional mobility in people with multiple sclerosis while also considering sex, clinical type of MS, and fall history. Additionally, this research aimed to examine the relationship between gait and balance in pwMS and the impact of falls on these two variables.

## 2. Materials and Methods

The research was conducted in the first half of 2024 at the Serbian Society for Multiple Sclerosis (SSMS). It involved people with MS (pwMS) who visited the Society during this period for various reasons, including membership card renewal, obtaining information about available therapies, accessing social benefits, and participating in programs organized by the Society. Membership in the SSMS is entirely voluntary.

The Serbian Society of Multiple Sclerosis focuses on improving the quality of life of individuals with MS through programs and services designed to support both patients and their families. Additionally, the SSMS plays a role in enhancing social participation and integration of pwMS. In accordance with the role of the SSMS and the research objectives, the general inclusion criteria for the participants were as follows: all participants were members of the Serbian Society of Multiple Sclerosis; they had been diagnosed with MS for more than ten years, with confirmation from a neurologist; participants were not younger than 20 or older than 70 years; they had experienced at least one fall in the six months preceding the research; they were able to communicate and understand the given instructions; and they provided voluntary informed consent to participate in the research. Participants independently completed the scales used in the research, while basic demographic data were obtained from the records maintained by the Serbian Society of Multiple Sclerosis.

The 12-item Multiple Sclerosis Walking Scale (MSWS-12) is a self-report measure that assesses the impact of MS on an individual’s walking ability, specifically evaluating self-perceived walking ability [22]. It is one of the most highly endorsed patient-reported outcome measures by MS-EDGE [23,24,25]. The scale evaluates various aspects of walking ability, including effort and speed, as well as tasks related to fall risk in pwMS [20], such as stair negotiation, standing, balance, ambulation support utilization, and attention span. PwMS who experience falls tend to have higher MSWS-12 scores than non-fallers [25]. Evidence suggests that this scale is effective in identifying individuals at risk of falling, particularly during remote consultations [26]. The MSWS-12 consists of 12 items, each scored on a scale of 1 to 5, where 1 indicates no limitation, and 5 represents an extreme limitation in gait-related tasks. A total score of 0 (equivalent to 12 summed points) signifies no impairment, whereas a score of 100 (equivalent to 60 summed points) represents the highest level of gait impairment [27]. This scale is a reliable and valid tool for assessing gait function in patients with MS [28].

The Activities-specific Balance Confidence (ABC) scale was used to assess balance. This scale was developed to evaluate an individual’s confidence in maintaining balance while performing daily activities. The ABC scale consists of 16 items covering a broad range of daily tasks, from less challenging to more challenging daily activities. Participants are asked to rate their confidence in performing each activity on a scale from 0% (not confident at all) to 100% (completely confident) in increments of 10%. The average score obtained serves as an indicator of balance confidence. A score above 80% reflects a high level of functioning, while scores between 50% and 80% indicate a moderate level of functioning. Scores below 50% suggest low levels of functioning, with scores below 67% specifically indicating a substantial risk of falling [29]. The ABC scale has demonstrated high reliability in patients with MS [30].

All statistical analyses were performed using SPSS (version 22; SPSS Inc., Chicago, IL, USA). Descriptive statistics (frequencies and percentages) and measures of central tendency (arithmetic mean and standard deviation) were used to analyze the demographic characteristics of participants and the basic indicators of the instruments applied. Pearson’s correlation analysis was conducted to examine the relationships between the MSWS-12 and ABC questionnaire scores and between these scores and fall history. Pearson’s correlation coefficients were interpreted as follows: 0.50–1.0 indicated a strong correlation, 0.30–0.49 a moderate correlation, and 0.10–0.29 a weak correlation [31]. To control for the potential influence of confounding variables on the relationship between the two primary variables, a partial correlation analysis was applied. An alpha level of 0.01 was set for all the statistical tests.

## 3. Results

The research sample consisted of 92 people with multiple sclerosis from the Republic of Serbia, aged between 22 and 69 years (M = 45.10; SD = 9.57). The sample included both sexes, with 82.6% female and 17.4% male participants, resulting in a female-to-male ratio of 4.75. The majority of participants were diagnosed with the relapsing-remitting subtype (56.5%), followed by primary-progressive (21.7%) and secondary-progressive (18.5%) subtypes. Additionally, 3.3% of pwMS did not specify their MS subtype. A total of 68 (73.9%) pwMS experienced at least one fall in the past six months. Based on previous studies, individuals were classified as fallers if they reported one or more falls during this period [16,32].

The research findings indicated that the overall sample had an average score of 58.17 (SD = 14.15) on the MSWS-12 and 89.8 (SD = 53.23) on the ABC scale. The theoretical range for the MSWS-12 is 12–60 points, while that for the ABC scale is 0–160 points. Statistically significant differences were observed on both scales in relation to MS clinical type and history of falls. The mean scores and statistical significance of the scales concerning sex, MS clinical type, and fall experience are presented in Table 1.

The correlation between balance confidence in performing daily activities, self-perceived walking ability, and certain demographic variables was determined using Pearson’s linear correlation coefficient (see Table 2). Preliminary analyses confirmed that the assumptions of normality, linearity, and homogeneity of variance were met. A strong negative correlation was found between the MSWS-12 and ABC scale (r = −0.76). Further analysis revealed that the correlation between the total scores on the MSWS-12 and ABC scale was r = −0.81 for men, although it was slightly lower for women (r = −0.75). For the relapsing-remitting subtype, the correlation was r = −0.72, while for the secondary-progressive subtype, it was r = −0.89, and for the primary-progressive subtype, it was r = −0.37. The correlations were marginally weaker in pwMS who had experienced a fall (r = −0.72) than in those who had not (r = −0.70). Subsequent analyses indicated that the calculated z-value for men and women was 0.64, while for pwMS who experienced a fall versus those who did not, the z-value was 0.41, demonstrating that the observed statistically significant differences were not random. Additionally, the z-value between individuals with RRMS and SPMS was −1.91, indicating that the statistically significant difference observed was not random.

In an additional analysis, we aimed to examine the relationship between the two variables (the total score on the MSWS-12 and ABC) while controlling for the potential influence of a third variable on this relationship. In this instance, the third variable was related to the falls experienced by pwMS. After removing the confounding variable, the results revealed a strong negative partial correlation between balance confidence in daily activities and self-perceived walking ability (r = −0.72). The zero-order correlation (r = −0.76) showed that excluding the confounding variable did not significantly change the strength of the relationship between the two variables.

## 4. Discussion

The aim of the research was to assess functional mobility and examine the relationship between gait and balance in people with MS, as well as the impact of falls on these two variables. The ABC and MSWS-12 scales represent an individual’s assessment of their capabilities across a range of tasks in relevant situations and are classified as patient-reported outcomes [21]. The ABC evaluates an individual’s confidence in maintaining balance during daily activities, whereas the MSWS-12 assesses the impact of multiple sclerosis on a person’s walking ability [27].

The results of the research showed that the average score on the MSWS-12 was 58.17 (14.15), and on the ABC was 89.8 (53.23). These results indicate moderate gait impairment and a high level of functioning. Our findings are consistent with those of Binshalan and colleagues [33], who reported a mean score of 56.9 ± 28.9 on the MSWS-12 in pwMS, and with those of Abate and colleagues [34], who found an average score of 54.01 ± 24.68 on the MSWS-12. In contrast, Garg and colleagues [35], who assessed a sample of 89 pwMS, found the mean score on the ABC to be 54.98 (29.46), while the mean score on the MSWS-12 was 62.55 (28.58).

Research has shown that men with MS may experience greater walking impairment than women, particularly as the disease progresses to the progressive phases. This may be related to the more severe course of disease in men. However, women may experience greater fatigue and muscle weakness, which can affect their balance and ability to walk. Fatigue, a common symptom of MS, can impact walking speed and endurance, leading to more frequent falls or difficulties in maintaining balance. Some studies have indicated that women with MS are more likely to experience disability in the upper limbs (due to demyelination of pathways), while men may show lower limb involvement, which directly impacts walking and balance [36,37].

An analysis of the data by sex, type of MS, and fall history indicates that individuals of both sexes, those with RRMS and those who have not suffered falls, report a high level of functioning and moderate gait impairment. The level of balance confidence was similar between men and women, although the female-to-male ratio was 4:1 (more precisely, 4.75). Our data are consistent with those of other studies [5,28].

Some research suggests that women may have better compensatory strategies for balance and gait than men, especially in the early stages of MS [9,38,39]. This could be due to factors like muscle recruitment patterns or a greater reliance on visual and sensory input to maintain balance. Men may experience a more pronounced loss of motor control earlier, which leads to more noticeable impairments in walking and balance [10]. Additionally, previous research has highlighted the influence of sex on functional capacity [40], mobility [41], and strength production [40]. These data help us better understand how the disease manifests depending on sex differences. Various studies have shown that walking and balance deficits are present even in individuals with RRMS who have no or only mild balance issues early in the disease [42]. People with SPMS and PPMS, as well as those who have experienced a fall, report the highest levels of gait impairment and moderate levels of functioning.

Age can have an impact on falls and functional mobility. The risk of falls increases for people over 65, so the combination of age and disease may lead to a higher risk of falls rather than the individual effects of these factors [19]. A higher likelihood of falls indicates impaired balance and walking difficulties.

Sosnoff and colleagues stated that 55.8% of participants in their research reported at least one fall in the previous 12 months, with a further 79% of these participants reporting two or more falls [19]. In our research, 73.9% of pwMS experienced falls in the past six months. Several studies have reported that pwMS have a substantial risk of falling [19,43], and walking activities are associated with an elevated risk of falls [9]. Impaired mobility is one of the most common and impactful consequences of MS [39]. Previous research has also revealed that fallers exhibit diminished balance confidence and greater self-perceived walking difficulty than non-fallers [35].

Perceptions of limitations in walking and balance confidence are closely related [35]. Our data show that the correlation between MSWS-12 and the ABC scale is strong and negative. Regarding the correlation between functional mobility, tested using the ABC and MSWS-12, and demographic variables, a statistically significant correlation was obtained between the type of MS and both the ABC and MSWS-12 scores, as well as between the MSWS-12 scores and those who experienced a fall. Some earlier studies that also used self-reports pointed out that balance disorders may correlate with an increased risk of falling [32].

In pwMS, primary impairments in muscle strength were observed in the lower extremities [44]. This is crucial, as the decline in lower limb strength adversely affects walking performance, balance, and the ability to ascend and descend stairs [45].

Some studies have indicated that older pwMS, who have slower walking speeds, poorer balance, and diminished walking endurance, are at an increased risk of falls. It is recommended that individuals who fulfill these criteria be closely observed for potential future falls [19].

The results of our research may have implications for the quality of life and social participation of pwMS. Impairment in functional mobility, especially walking, affects the independence of these patients. In other words, walking problems and issues with balance confidence have a strong negative impact on the quality of life of people with multiple sclerosis, not only in terms of motor functions but also in relation to participation in social activities [46], activities of daily living, and employment [47,48]. Ponzio et colleagues state that difficulties related to movement, coordination, or balance impairment, dizziness, problems with prolonged standing, getting easily tired, bowel problems, and fatigue were the most significant barriers to work activities and employment [49]. Vocational rehabilitation and social support can play crucial roles in pwMS who have mobility disorders.

We acknowledge some limitations of this research. For example, only subjective tests were used, specifically self-rating scales/self-reported measures of functional mobility in people with MS. They should be regularly assessed using clinical and laboratory-based analysis tools. In other words, objective clinical assessments, such as functional tests or gait analysis, are necessary to confirm the robustness of the data and draw stronger conclusions. Second, the duration of the disease, EDSS, and medical comorbidities were not taken into account, and these could have had some impact on the results. Finally, we would like to point out the sample size as a limitation. We believe that future research should include a larger sample so that more generalized conclusions can be drawn.

## 5. Conclusions

MS is a disease that presents many challenges. In the Republic of Serbia, the number of pwMS referred for rehabilitation and other therapies is steadily increasing. According to data from the MS International Federation, 96% of pwMS report that they can access rehabilitation or other therapies for difficulty walking, 93% for stiffness and spasms, 50% for tremors, and only 21% for heat sensitivity [2]. Addressing balance and walking issues in MS requires a multidisciplinary approach that combines physical therapy, medication, assistive devices, and exercise programs. Regular assessments are crucial for monitoring changes in mobility, allowing timely adjustments to treatments that can improve the patient’s quality of life. Overall, balance and gait problems in patients with MS significantly increase the risk of falls, which can have a detrimental impact on their quality of life. Timely and appropriate rehabilitation is essential for reducing this risk and enhancing patient functionality.

This research found that pwMS had partially preserved functional mobility and that mobility was not significantly impacted by whether the individual had experienced a fall. The results can be used to design suitable treatments and support programs for these individuals. For example, some clinical practice suggestions are as follows: Individualized rehabilitation programs: Given the moderate gait impairment and high level of functionality, therapy techniques should be customized to each patient’s abilities, with a focus on preserving and enhancing mobility. Balance Training: Since there is a link between balance confidence and walking ability, rehabilitation programs should incorporate exercises aimed at improving postural control and proprioception. Psychological support: Cognitive-behavioral therapy and patient education on fall prevention strategies can help boost self-confidence and improve balance maintenance. Physical activity and adaptive exercises: Regular physical activity, including walking, swimming, and Pilates, are recommended to maintain functional mobility and reduce the risk of further gait impairments. Regular follow-up for pwMS: it is essential to periodically conduct functional assessments to detect changes in mobility and adjust therapeutic approaches as necessary.

As future research directions, we propose conducting a longitudinal study to assess how functional mobility and balance confidence evolve over time in people with different forms of multiple sclerosis. We also propose investigating how anxiety, depression, and fear of falling influence walking ability and balance in pwMS. Additionally, we propose the development and testing of digital tools, wearable sensors, and applications for monitoring gait and balance in real-world environments. Further research should also explore how different rehabilitation strategies (e.g., vestibular therapy, strength training, and virtual reality) affect gait and balance confidence in people with multiple sclerosis. Finally, future research should compare the effectiveness of various physical activities (e.g., yoga, hydrotherapy, functional training) to determine which approaches provide the best outcomes for preserving functional mobility.

## Figures and Tables

**Table 1:** The average score of the scales in relation to sex, clinical type of MS, and the experienced fall.

	ABC (M, SD)	*p*	MSWS-12 (M, SD)	*p*
Sex				
Male (N = 16)	92 (57.39)	0.859	60 (15.36)	0.750
Female (N = 76)	89.38 (52.71)		57.91 (13.98)	
Clinical type of MS				
RRMS (N = 52)	111.63 (48.25)		48.72 (12.18)	
SPMS (N = 17)	59.12 (50.68)	0.000	76.12 (13.17)	0.000
PPMS (N = 20)	58.60 (37.33)		70.83 (11.55)	
Not specify (N = 3)	94.33 (74.74)		38.88 (8.08)	
Fall				
Yes (N = 68)	77.44 (50.48)	0.000	63.7 (13.18)	0.000
No (N = 24)	124.96 (45.21)		42.92 (12.90)	

Note: RRMS—Relapsing-remitting multiple sclerosis; SPMS—Secondary-progressive multiple sclerosis; PPMS—Primary-progressive multiple sclerosis.

**Table 2:** Correlation between functional mobility and certain demographic variables.

	Sex	Clinical Type of MS	Fall
MSWS-12	−0.034	0.321 **	−0.389 **
ABC	−0.019	−0.389 **	0.394

** Correlation is significant at the 0.01 level.

## Data Availability

The raw data supporting the conclusions of this article will be made available by the authors upon request.

## Abbreviations

The following abbreviations are used in this manuscript:

pwMS	People with Multiple Sclerosis
MS	Multiple Sclerosis
MSWS-12	The 12-item Multiple Sclerosis Walking Scale
ABC	The Activities-specific Balance Confidence
SSMS	Serbian Society of Multiple Sclerosis
